# Long-term Kinetics of Vibriocidal Antibody Responses After *Vibrio cholerae* Infection in the Democratic Republic of Congo

**DOI:** 10.1093/infdis/jiaf382

**Published:** 2025-07-22

**Authors:** Kilee L Davis, Carmen Nieznanski, Felicien M Maisha, Ben J Brintz, Christy H Clutter, Meer T Alam, Cyrus Saleem, Afsar Ali, J Glenn Morris, Daniel T Leung

**Affiliations:** Division of Infectious Disease, Department of Internal Medicine, University of Utah, Salt Lake City; Division of Infectious Disease, Department of Internal Medicine, University of Utah, Salt Lake City; Department of Sociology, College of Humanity and Social Sciences, University of Goma, Democratic Republic of Congo; Emerging Pathogens Institute, University of Florida, Gainesville; Division of Epidemiology, Department of Internal Medicine, University of Utah, Salt Lake City; Division of Infectious Disease, Department of Internal Medicine, University of Utah, Salt Lake City; Emerging Pathogens Institute, University of Florida, Gainesville; Emerging Pathogens Institute, University of Florida, Gainesville; Emerging Pathogens Institute, University of Florida, Gainesville; Emerging Pathogens Institute, University of Florida, Gainesville; Division of Infectious Disease, Department of Internal Medicine, University of Utah, Salt Lake City

**Keywords:** antibody kinetics, cholera, seroepidemiology, vibriocidal

## Abstract

Interpretation of seroepidemiology studies of cholera relies on knowledge of antibody kinetics, which are not well known in African populations. We performed vibriocidal antibody assays on 212 serum samples from 115 patients with culture-positive cholera (median age, 8 years) in Goma, Democratic Republic of Congo, which were collected at enrollment and 3 to 449 days after. Vibriocidal responses peaked at 7 to 40 days after symptom onset, with 89.5% waning to a titer ≤160 by 180 days. We used a bayesian exponential decay model to show an 88% probability of the posterior distribution supporting a faster decay in children ≤5 years of age.

## Teaser

Antibody kinetics are important for interpretation of seroepidemiology studies of cholera. We describe the long-term vibriocidal kinetics of patients with cholera from Goma, Democratic Republic of Congo, and show that children ≤5 years of age may have faster decay as compared with older persons.

Cholera is an acutely dehydrating diarrheal disease caused by the aquatic gram-negative toxigenic bacterium *Vibrio cholerae* [1]*. V cholerae* is transmitted via the ingestion of contaminated food and/or water, making it most prevalent in poverty-afflicted communities around the world where water and sanitation infrastructure is limited. Surveillance for cholera can be challenging due to limited capacity for disease surveillance in places where the disease occurs, variations in its seasonality, and the nonspecificity of cholera symptoms [2]. An emerging method for estimating cholera burden is serosurveillance, involving measurement of cholera-specific antibody in blood obtained from a representative sampling of a defined population [3]. Aside from burden estimates, serosurveillance can be used to estimate a population's immunity level. Such knowledge can inform public health strategies, including the timing of deployment of interventions such as vaccination.

Longitudinal studies examining kinetics of antibody responses to cholera are crucial for interpreting cross-sectional serosurveillance data. Until now, kinetics of cholera antibody response have been mostly limited to patients with severe cholera in Bangladesh (a hyperendemic region) [4]. There is a paucity of data on the kinetics of antibody responses against cholera in endemic regions outside of South Asia. Our primary aim is to investigate the long-term kinetics of *V cholerae* antibody responses among patients in Goma, Democratic Republic of Congo (DRC), a cholera-endemic region in Africa. Our secondary aim is to compare the antibody kinetics between young children and older persons.

## METHODS

### Patient Recruitment and Collection of Samples

To examine longitudinal antibody responses to cholera, we measured vibriocidal antibody titers of patients with culture-confirmed *V cholerae* O1 infections. Study participants were recruited from patients with acute watery diarrhea who presented to cholera treatment centers operated by the health division of the province of North Kivu in Goma, DRC, from August 2020 to October 2023. Written informed consent was obtained from participants or their parents/guardians for those aged ≤17 years, with assent from participants aged ≥7 years. Following informed consent, clinical and epidemiologic data were collected into REDCap [5] through the University of Florida Clinical and Translational Science Institute. This study was approved by the institutional review board of the University of Florida and the Comité Éthique de l’Université Libre des Pays des Grands Lacs.

Stool and venous blood samples were collected at enrollment. Stool cultures were performed to detect *V cholerae* O1. Serum was separated and stored at −80 °C for future analysis. Two follow-up visits were attempted to collect venous blood samples at approximately 4 weeks and 6 months postenrollment. Time points were used to generally describe serum sample collection, while the exact day since symptom onset was used in our analysis.

### Vibriocidal Assay

Vibriocidal antibodies are the best correlate of protection against cholera [6] and the best marker for prior infection [4]. While the vibriocidal assay has been used for cholera research since the 1960s, its application in population-based surveillance studies has become more common in recent years [7–9]. We performed the vibriocidal assay for *V cholerae* O1 Inaba (T19749) and Ogawa (X25049) serotypes as previously described [4] (Supplemental Methods). Samples below the detection limit were assigned a titer of 5, and those above the limit were assigned a titer of 20,480.

### Statistical Analysis

We examined the kinetics of vibriocidal titers over time, using the titer of the serotype matched by culture results. We used the *ggplot2* package in R (version 4.4.1) for data visualization.

Given the paucity of data on the long-term kinetics of cholera antibody responses in children ≤5 years old, we used vibriocidal titers from participants who had at least 2 paired samples to fit a bayesian model. We also used the No U-turn Sampler in the *cmdstanr* package in R to estimate exponential decay parameters describing the amplitude of titers and the rate of decay. The model assumes that the observed and logged titer values follow a normal distribution with mean $N_{0(i)}*e^{-(k1+over_{i}*k2)}$ and variance *σ*, where $N_{0(i)}$ is the person-specific amplitude, k1 is the decay rate for the group ≤5 years old, $over_{i}$ is an indicator variable for being >5 years old, and k2 is the change in decay rate for the group >5 years old. We fit an additional model to test for a biphasic decay with an alternative mean: $N_{0(i)}*e^{-(k1*t)}*e^{-(k2*(t-\tau )*I(t>\tau ))}$, where I(t>τ)=1 if t>τ; otherwise, I(t>τ)=0. Given that in preliminary analyses, the majority of patients’ initial vibriocidal titer was their highest titer, we assumed that we missed the initial rise in titer value to the amplitude; thus, our model equation does not include the rise in titer value and examined only the rate of decay.

We fit all models using 4 independent chains, each run for 2000 iterations (1000 warm-up and 1000 sampling) with uninformative priors. Convergence was assessed by examining that no iterations triggered divergences or exceeded the maximum tree depth, by confirming the Gelman-Rubin statistic for all parameters were <1.01, and by ensuring that effective sample sizes were sufficiently large (>200).

To examine the epidemiologic relevance of the difference in decay kinetics between age groups, we estimated the average titer value at 180 days, using a model that allows a different decay rate for participants >5 years old and a model that assumes equal decay regardless of age. We used a threshold vibriocidal titer ≥160 as an indicator of prior infection to establish and compare burden at 6 months postexposure between models.

## RESULTS

**Figure 1.**
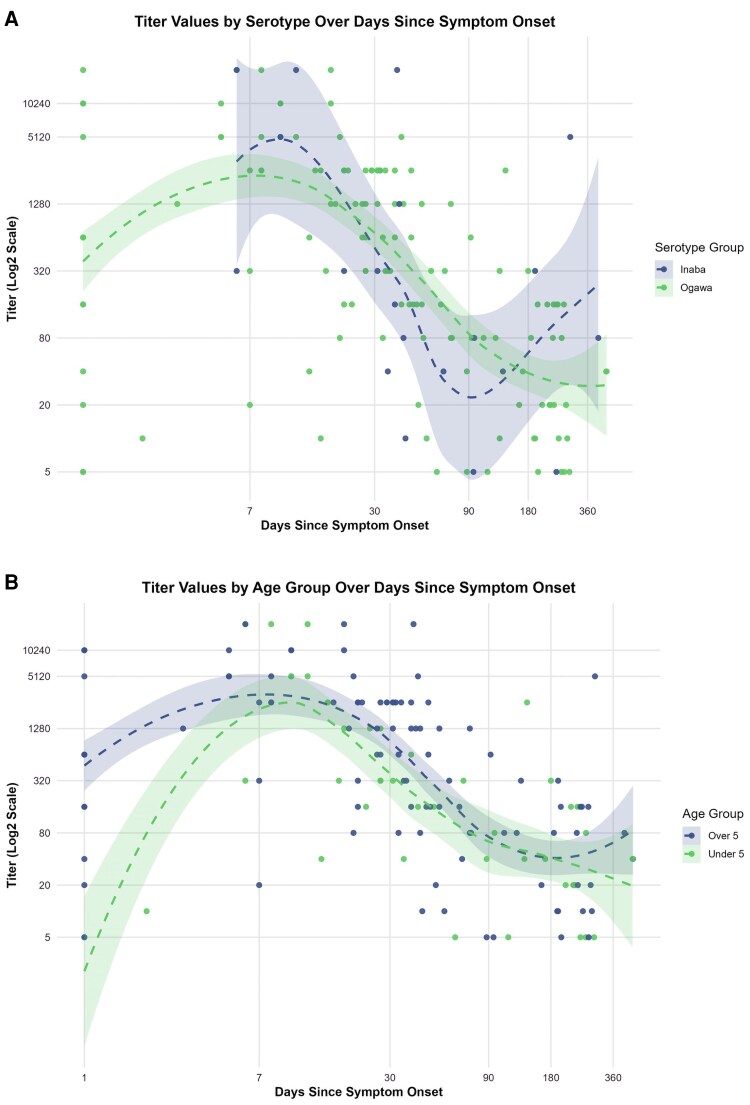
Vibriocidal titer responses over time from culture-confirmed cholera cases. Vibriocidal titers by matching *Vibrio cholerae* O1 serotype from 101 participants with serotype information available, by (*A*) serotype and (*B*) age group, plotted on log_2_-transformed scales. Hashed lines are LOESS smoothed data with 95% CIs in shade. LOESS, locally estimated scatterplot smoothing.

We performed vibriocidal assays on serum from 212 samples from 115 patients with culture-confirmed *V cholerae* infection (53% male; median age, 8 years [IQR, 4–18; range, 1–76]). Of the 101 participants for which serotype information was available, 83 (82%) were infected with Ogawa serotype, 15 (15%) with Inaba, and 3 (3%) with Hikojima. Children ≤5 years old represented 37% of the participants, and 27 (25%) self-reported prior oral cholera vaccination. Twenty (20%) participants had serum samples from 3 time points, 57 (56%) had 2 time points, and 38 (38%) had 1 time point (Supplementary Table 1). Sample collection from many households was delayed due to unanticipated problems in reaching patient homes for repeat visits and restrictions on travel related to safety concerns. Because of this, there was substantial variability in the time of collection of what were initially scheduled as 4-week and 6-month postinfection visits. Consequently, data were recorded and analyzed as the number of days between the onset of illness and the date of sample collection, with second samples collected between 3 and 297 days and third samples collected between 25 and 449 days after symptom onset (Figure 1).

**Figure 2.**
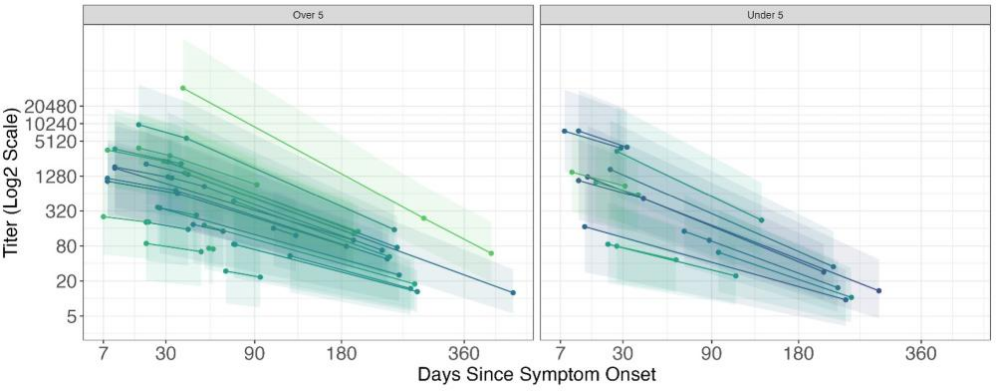
Comparison of vibriocidal decay kinetics between age groups. Estimated average titer value and 95% posterior credible interval for serotype-matched vibriocidal titers from the exponential decay equation plotted against days since symptom onset, by participants aged >5 years (left) and ≤5 years (right). Each line represents the vibriocidal titer decay of a participant with at least 2 paired samples, fitted to a bayesian model to estimate exponential decay parameters describing the amplitude of titers and the rate of decay.

Overall, the highest vibriocidal responses were noted between 7 and 40 days and waned over time, with 92.7% of samples waning to a titer ≤320 by 90 days and 89.5% waning to a titer ≤160 by 180 days (Figure 1, Supplementary Figure 1). We then used data from all participants who had at least 2 paired samples to examine differences in decay kinetics between participants ≤5 years of age and those >5 years. After exclusion of those with titers below the limit of detection between 3 and 60 days, the final analysis included 39 participants: 26 aged ≤5 years and 13 aged >5 years (Figure 2). In the exponential decay model, we found a negative posterior mean for $k_{2}$, the parameter indicating a change in decay for the participants >5 years old with 95% posterior credible intervals overlapping 0 ($k_{2}$, −0.0012; −0.0030 to 0.0004). The draws from the posterior distribution of *k*_2_ suggest that there is an approximately 88% probability of a slower decay in participants >5 years; that is, 88% of the posterior distribution is <0. Given that prior vaccination may increase the probability of a slower decay, we performed a sensitivity analysis among those without prior vaccination (28 participants representing 66 time points) and found similar results ($k_{2}$, −0.0014; −0.0041 to 0.0007), with an approximately 83% probability of a slower decay in participants >5 years old. In the biphasic decay model, we estimate an initial decay of $k_{1}$ (0.0094; 0.0057–0.00139) and found evidence of a positive $k_{2}$ (0.0017; 0.0007–0.0026), suggesting a slower decay following a median 62 (IQR, 37-90) days since symptom onset. We found that all fit models achieved convergence.

We examined the impact that a difference in decay kinetics would have on estimation of disease burden by estimating the average titer value at 180 days (approximately 6 months) using a model with and without differential kinetics by age group. Assuming that a vibriocidal threshold of 160 indicates prior infection, we found that not using a model with the differential kinetics resulted in the classification of an additional 21% of children ≤5 years old as infected.

## DISCUSSION

In this longitudinal study of patients with culture-confirmed cholera in Goma, DRC, we describe the vibriocidal antibody kinetics of patients from a cholera-endemic area in Africa for up to 1 year after symptom onset. Our analysis suggests that vibriocidal antibody responses from children ≤5 years old may wane more rapidly than those in older children and adults and that both groups had few with titers >160 at 1 year postinfection. These data suggest that young children may be more vulnerable to repeat infection than adults, although both groups may require vaccination to boost immunity.

The predominance of young children in our study is unique when compared with prior studies of vibriocidal kinetics, which have featured mostly older children and adults. Leveraging this and the longitudinal nature of the study, we show that the waning of the vibriocidal response in young children (age ≤5 years) is likely faster than that of older persons. While the 95% posterior credible interval contains 0, a null result in the frequentist sense, our bayesian model suggests an 88% posterior probability of differing decay rates between age groups, which translates to a 21% difference in the classification of prior infection in children ≤5 years old. This finding fills a knowledge gap, suggesting that interventions such as the use of vaccination to boost immune response may benefit even those in this age group who had a recent infection.

We found that the vibriocidal antibody response decays over time in a biphasic manner—results that are consistent with a previous study of vibriocidal kinetics in medically attended severe cholera cases in Bangladesh [4]. Due to differences in laboratories, assay controls, and sampling approach, we are unable to make direct statistical comparisons in the magnitude of responses between studies. Nevertheless, factors that may contribute to differences in vibriocidal titer among populations from different geographic regions include degree of “endemicity,” the proportion with prior vaccination, and age distribution.

Our findings underscore the value of longitudinal serologic data to inform estimation of burden and guide the design of future serosurveys, which may in turn inform public health interventions such as WASH interventions (water, sanitation, and hygiene) and vaccination campaigns. In particular, evidence of more rapid antibody waning in young children could support age-stratified serosurveys and intervention strategies.

Our study had several limitations. Study participants were limited to individuals who could access cholera treatment centers. The small sample size reduced statistical power, particularly for subgroup analyses such as vaccinated vs unvaccinated individuals. More broadly, the limited number of participants introduces uncertainty (ie, wider posterior intervals) into our bayesian modeling estimates, including comparisons of antibody decay rates across age groups. Larger studies are needed to confirm these preliminary findings. Nevertheless, this represents one of the largest longitudinal cohorts examining kinetics of vibriocidal responses in young children, informing the design and interpretation of future studies. Although the vibriocidal response is the best nonmechanistic correlate of protection against *V cholerae* [6], the vibriocidal titer alone has not demonstrated to be completely predictive of long-term immunity against clinical disease [10].

Nevertheless, we present the postinfection vibriocidal kinetics of a large cohort of patients with culture-confirmed cholera in an endemic region of Africa and show evidence that younger children may have a faster decay rate than older persons. Our study begins to address the data gap in longitudinal antibody response studies outside of South Asia and suggests that more frequent boosting with vaccine may be needed for cholera-vulnerable communities such as those in Goma, DRC.

## Supplementary Material

jiaf382_Supplementary_Data

## Supplementary Data

Supplementary materials are available at *The Journal of Infectious Diseases* online (http://jid.oxfordjournals.org/). Supplementary materials consist of data provided by the author that are published to benefit the reader. The posted materials are not copyedited. The contents of all supplementary data are the sole responsibility of the authors. Questions or messages regarding errors should be addressed to the author.

## Notes

***Acknowledgments.*** We acknowledge the contributions of the DRC National Program for the Elimination of Cholera and Other Diarrheal Diseases, the North Kivu Provincial Health Division, Appui Medical Integre aux Activites de Laboratoire, and HEAL Africa.

***Author contributions.*** A. A., J. G. M., and D. T. L. conceived and planned the experiments and acquired funding for the study. F. M. M. and M. T. A. recruited and enrolled patients and collected samples for the study. K. L. D. and C. N. carried out the laboratory assays. K. L. D., C. N., B. J. B., C. H. C., and C. S. performed data management and statistical analysis. K. L. D., C. N., and D. T. L. wrote the first draft of the manuscript. All authors reviewed and edited the manuscript.

***Data availability*.** Code for the analysis is deposited at https://github.com/LeungLab/Vibrio-Kinetics.

***Financial support.*** This work was supported by the National Institutes of Health (R01AI135115 to D. T. L., R01AI138554 to J. G. M.); the University of Utah Undergraduate Research Opportunities Program (to C. N.); and the Dr Thomas D. Rees and Natalie B. Rees Presidential Endowed Chair in Global Medicine (to D. T. L.).
