# Supplementary material for: Long-term Kinetics of Vibriocidal Antibody Responses After Vibrio cholerae Infection in the Democratic Republic of Congo
Source: J Infect Dis. 2025 Jul 22;232(4):e621–5. doi: 10.1093/infdis/jiaf382 (PMC12526859; doi:10.1093/infdis/jiaf382)
Supplement: jiaf382_Supplementary_Data [file jiaf382_supplementary_data.docx]

**Supplemental Materials**

**Supplemental Methods**

Vibriocidal Assay

We performed the vibriocidal assay for *V. cholerae* O1 Inaba (T19749) and Ogawa (X25049) serotypes as previously described [4]. A positive control, an IgG monoclonal antibody against *V. cholerae* O1 O-specific polysaccharide (gift of Dr. Jason Harris, Massachusetts General Hospital, Boston MA), was added to naïve serum at 6.24 µg/µL corresponding to a titer of 640. Heat-inactivated serum samples were duplicated, then serially diluted two-fold in saline from 1:10 to 1:10,240 in a 96-well microplate. Negative control (bacteria without serum) and positive control were included on each plate. *V. cholerae* O1 Ogawa (X25049) and Inaba (T19749) strains were grown in Luria Broth (LB) at 37°C for 3 hours, washed with saline, then resuspended to an optical density at 600nm (OD600) of 0.3. A Growth Indicating Solution was prepared with guinea pig complement, diluted 1:10 and the bacterial suspension diluted 1:20 in saline. This solution was added to the samples and positive control wells. Plates were incubated at 37ºC at 50 rpm for 1 hour, then LB added and incubated at 37ºC without shaking for 2 hours. Plates were measured when the growth indicator reached OD600 of at least 0.2. Vibriocidal titers were defined as the reciprocal of the highest serum dilution resulting in approximately 50% of the mean OD600 of the Growth Control wells.

**Supplemental Table 1. Summary of patient and sample counts by serotype.**

| Serotype Group | Total # of Patients | # with 1 sample | # with 2 samples | # with 3 samples | Total Samples |
| --- | --- | --- | --- | --- | --- |
| Ogawa | 83 | 26 | 40 | 17 | 157 |
| Inaba | 15 | 4 | 10 | 1 | 27 |
| Hikojima | 3 | 2 | 1 | 0 | 4 |
| No Serotype Available (NA) | 14 | 6 | 6 | 2 | 24 |
| Totals | **115** | **38** | **57** | **20** | **212** |

**Supplemental Figure 1. Individual vibriocidal titer kinetics by serotype.** Vibriocidal titers by matching *V. cholerae* O1 serotype from participants with serotype information available, with lines connecting samples from individual participants, plotted on log2-transformed scales. Hashed lines are LOESS smoothed data with confidence intervals in shade.

**
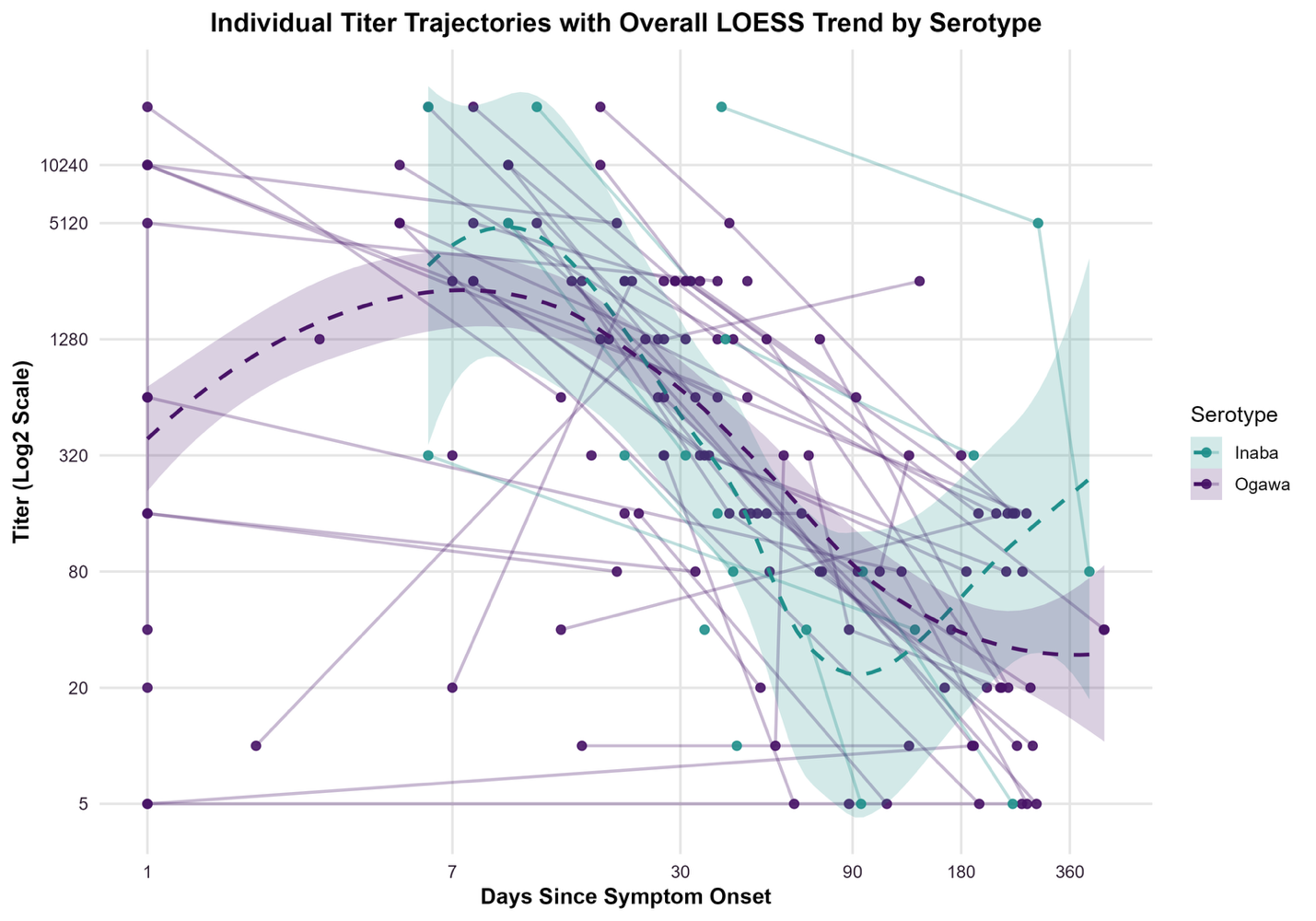
**
